# A new gentle reduction technique for patients with congenital diaphragmatic hernia—A case report

**DOI:** 10.1016/j.ijscr.2019.03.001

**Published:** 2019-03-06

**Authors:** Takafumi Kawano, Oliver J. Muensterer

**Affiliations:** Department of Pediatric Surgery, University Medicine Mainz, Germany

**Keywords:** Diaphragmatic hernia, Hernia sack, Reduction, Twist

## Abstract

Reducing the herniated viscera such as the spleen, the liver, and the bowel in thoracoscopic repair of congenital diaphragmatic hernia can be challenging. The small instruments involved can easily create damage, particularly to the friable capsule of the solid organs involved. We herein demonstrate a new gentle method of reducing the diaphragmatic hernia using sequential twisting of the sac. The patient was a 3 day old girl with left sided Bochdalek diaphragmatic hernia. Chest X-ray showed intestinal herniation into the thoracic cavity, and we planned to perform elective Minimal-invasive surgery.

Once in the chest, the fundus of the hernia sac is grasped with a blunt endosurgical grasper. The grasper is then turned, twisting the sac around the shaft of the instrument and thereby reducing the hernia content. Once maximal reduction with one hand is achieved, the twisted tissue is grasped with the contralateral instrument and the maneuver repeated until the entire hernia content is reduced. Subsequently, the sac is removed circumferentially using the monopolar electrocautery hook. The diaphragm is then closed with interupted figure-eight sutures of 2-0 silk.

The operative time was 65 min, and there were no intraoperative complications. She was discharged home on postoperative day 5. Half a year later, she was asymptomatic without any signs of recurrence. In conclusion, the twisting technique allows for gentle, gradual reduction of diaphragmatic hernias with a sac without injury to the herniated viscera.

## Highlights

• The number of cases which can undergo thoracoscopic repair for congenital diaphragmatic hernia is limited.
• Reducing the herniated viscera in thoracoscopic repair of CDH can be dangerous due to their vulnerability of neonatal organs.
• This twisting technique allows for gentle reduction of diaphragmatic hernias with a hernia sac, without a risk of injury.

## 1 Introduction

Congenital diaphragmatic hernia (CDH) is a life-threatening disorder which needs definitive surgical repair. Traditionally, laparotomy or thoracotomy was the standard procedure for this disease, but the number of cases performed by minimal-invasive surgery (MIS) for neonatal CDH has increased since the first laparoscopic repair of children with CDH was reported [1]. Nowadays, the thoracoscopic approach in newborn is widely used in specialized centers around the world [2,3].

However, endoscopic surgery is somewhat limited by varying degrees of severity of the associated pulmonary hypertension. Pediatric surgeon, therefore, do not have the opportunity to perform many cases of thoracoscopic repair of CDH, which can reduce their readiness and familiarity with the procedure, especially in pediatric surgery trainees. There are many pitfalls of the technique. Among of them, there is a certain vulnerability of the herniated neonatal organs such as the spleen, liver and bowel while being handled with small endosurgical instruments. We therefore demonstrate a new gentle and easy method of reducing the diaphragmatic hernia using sequential twisting technique of the sac of CDH, making direct grasping and manipulating of the delicate viscera unnecessary.

## 2 Case report

**Fig. 1:**
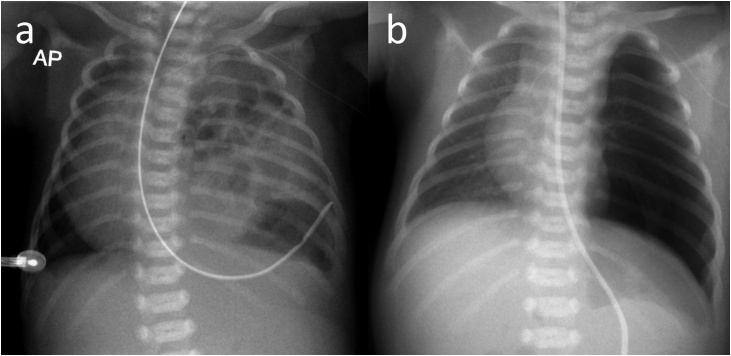
A) Preoperative image: Chest X-ray showed intestinal herniation into the thoracic cavity. B) Postoperative image.

**Fig. 2:**
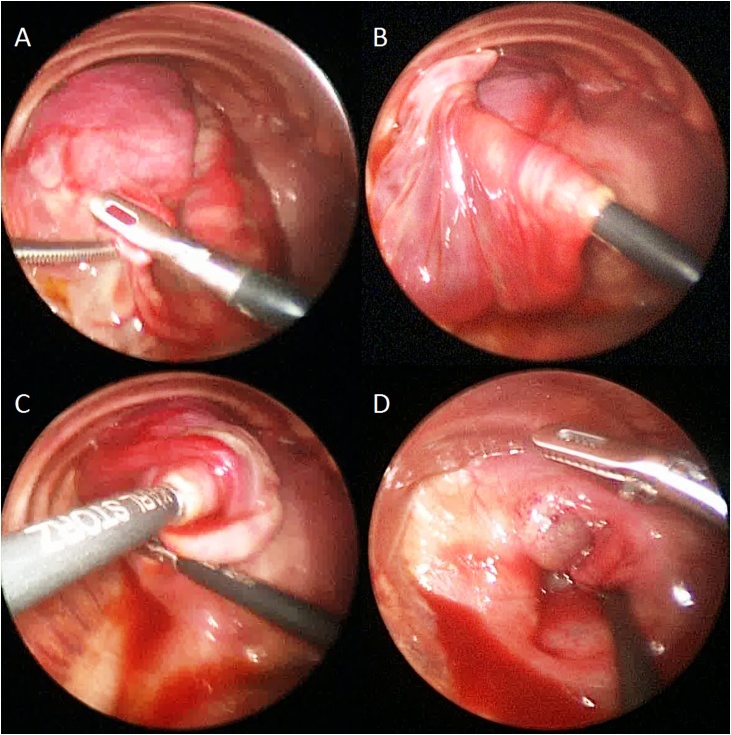
The procedure of twisting technique. A) The fundus of the hernia sac is grasped with a blunt endosurgical grasper. B–D) The grasper is turned, twisting the sac around the shaft of the instrument and thereby reducing the hernia content. Once maximal reduction with one hand is achieved, the twisted tissue is grasped with the contralateral instrument and the maneuver repeated until the entire hernia content is reduced.

The patient was a 3 day old girl with left sided Bochdalek diaphragmatic hernia diagnosed prenatally. She was born at full term through vaginal delivery. Chest X-ray showed intestinal herniation into the thoracic cavity (Fig. 1A). After intubation and ventilation, we planned to perform elective MIS repair of the hernia. The patient was placed in a right lateral position, and three trocars were inserted (3 mm, middle axillary fourth intercostal space (ICS) for the endoscope; 3 mm, anterior axillary fifth ICS for the operator’s right hand; 3 mm, posterior axillary fifth ICS for the operator’s left hand). Artificial capnothorax by CO_2_ inflation (5 mm Hg, 1 L/min) was established. Under inspection using a 3-mm 30 degrees endoscope, a hernia sac was found. Instead of initially resecting the hernia sac, we decided to use it to reduce the herniated organs. This was accomplished by grasping the fundus of the hernia sac and twisting it around the instrument. Once maximal reduction with one hand was achieved, the twisted tissue was grasped with the contralateral instrument and the maneuver repeated until the entire hernia content was completely reduced. (Fig. 2) Subsequently, the sac was removed circumferentially using the monopolar electrocautery hook. The diaphragm was then closed with interrupted figure-eight sutures of 2-0 silk. The operative time was 65 min, and there were no intraoperative complications (Video, Fig. 1B). She was discharged home on postoperative day 5. Half a year later, she was asymptomatic without any signs of recurrence.

## 3 Discussion

In 1995, van der Zee and Bax reported a 6-month-old child CDH patient treated by laparoscopy [1]. Few years later, Becmeur et al. reported thoracoscopic repair in three pediatric patients with late-presenting CDH [4]. Since then, MIS has been becoming a commonly used approach for repair of CDH in stable selected neonatal cases all over the world.

Up to now, many studies have been reported about advantages of MIS for thoracoscopic CDH repair. It has been suggested to reduce the duration of postoperative mechanical ventilation, the need for narcotics on the short term, and the incidences of subsequent scoliosis, chest deformation, shoulder muscle girdle weakness, and small bowel obstruction. On the other hand, MIS was associated with recurrence rate [[5], [6], [7]]. However, all these corresponding studies were based on retrospective data, with potential for selection bias and heterogeneity concerning the numerous differences in techniques, surgeon experience, and perioperative management. Some cases may have simply been associated with technically more demanding findings, and thereby may have been prone to recurrence. According to the survey of International Pediatric Endosurgery Group (IPEG), a group of pediatric endosurgery experts, as many as half of respondents performed less than 5 cases in a year. In addition, only 24% surgeons did more than 10 cases a year [8]. The number of cases which can undergo thoracoscopic repair is limited, and it can take times for pediatric surgeons to acquire the skills of thoracoscopic repair for CDH. At this point, the twist technique is easy to perform and can even be performed by pediatric surgery trainees, such as in our case.

There are 2 main parts of the procedure, which are reduction of abdominal viscera and diaphragm repair. Among of them, reducing the herniated viscera such as the spleen, the liver, and the bowel in thoracoscopic repair of CDH can be dangerous due to their vulnerability of neonatal organs. Some studies have reported bleeding, solid organ laceration, and gastrointestinal perforation as intraoperative complications during thoracoscopic CDH repair [9]. The small rigid instruments involved can easily cause damage, particularly to the fragile capsule of the solid organs being reduced. Our new twisting technique can prevent such complications Postoperative small bowel obstruction (SBO) has been reported as high as 20% due to intestinal adhesion after CDH open repair. Thoracoscopy for CDH may be associated with a lower rate [10]. We hypothesize that the rate of postoperative SBO may be even further reduced by the twisting technique, since the small bowel is not directly manipulated.

The limitation of our technique is that it is feasible only for CDH cases with a hernia sac. Also, it does not allow for direct inspection of the organs prior to reduction.

## 4 Conclusion

The new twisting technique allows for gentle, gradual reduction of diaphragmatic hernias with a hernia sac, without a risk of injury to the herniated viscera.

## Conflicts of interest

None.

## Sources of funding

This research did not receive any specific grant from funding agencies in the public, commercial, or not-for-profit sectors.

## Ethical approval

This study is a case report and does not require ethical approval in its current form.

## Consent

Written informed consent was obtained from the patient for publication of this case report and accompanying images. A copy of the written consent is available for review by the Editor-in-Chief of this journal on request.

## Author’s contribution

T. Kawano drafted the article and approved final version. O. J. Muensterer revised drafted article and approved final version.

## Registration of research studies

This case report does not require registration as a research study.

## Guarantor

The Guarantor is Professor O.J. M.

## Provenance and peer review

Not commissioned externally peer reviewed.

## References

[1] van der Zee D.C., Bax N.M. (1995). Laparoscopic repair of congenital diaphragmatic hernia in a 6-month-old child. Surg. Endosc..

[2] Schneider A., Becmeur F. (2018). Pediatric thoracoscopic repair of congenital diaphragmatic hernias. J. Vis. Surg..

[3] Fujishiro J., Ishimaru T., Sugiyama M., Arai M., Suzuki K., Kawashima H. (2016). Minimally invasive surgery for diaphragmatic diseases in neonates and infants. Surg. Today.

[4] Becmeur F., Jamali R.R., Moog R., Keller L., Christmann D., Donato L. (2001). Thoracoscopic treatment for delayed presentation of congenital diaphragmatic hernia in the infant. Surg. Endosc..

[5] Putnam L.R., Gupta V., Tsao K., Davis C.F., Lally P.A., Lally K.P. (2017). Factors associated with early recurrence after congenital diaphragmatic hernia repair. J. Pediatr. Surg..

[6] Szavay P.O., Obermayr F., Maas C., Luenig H., Blumenstock G., Fuchs J. (2012). Perioperative outcome of patients with congenital diaphragmatic hernia undergoing open versus minimally invasive surgery. J. Laparoendosc. Adv. Surg. Tech..

[7] Cho S.D., Krishnaswami S., Mckee J.C., Zallen G., Silen M.L., Bliss D.W. (2009). Analysis of 29 consecutive thoracoscopic repairs of congenital diaphragmatic hernia in neonates compared to historical controls. J. Pediatr. Surg..

[8] Lacher M., St Peter S.D., Laje P., Harmon C.M., Ure B., Kuebler J.F. (2015). Thoracoscopic CDH repair—a survey on opinion and experience among IPEG members. J. Laparoendosc. Adv. Surg. Tech..

[9] Vijfhuize S., Deden A., Costerus S., Sloots C., Wijnen R. (2012). Minimal access surgery for repair of congenital diaphragmatic hernia: is it advantageous?—an open review. Eur. J. Pediatr. Surg..

[10] Putnam L.R., Tsao K., Lally K.P., Blakely M.L., Jancelewicz T., Lally P.A. (2017). Minimally invasive vs open congenital diaphragmatic hernia repair: is there a superior approach?. J. Am. Coll. Surg..

